# Correction: Sonochemical synthesis of polyoxometalate-stabilized gold nanoparticles for point-of-care determination of acetaminophen levels: preclinical study in an animal model

**DOI:** 10.1039/d0ra90056g

**Published:** 2020-05-12

**Authors:** Tahereh Rohani Bastami, Abolphazl Ghaedi, Scott G. Mitchell, Aida Javadian-Saraf, Mohammad Karimi

**Affiliations:** Department of Chemical Engineering, Quchan University of Technology Quchan 94771-67335 Iran t.rohani@qiet.ac.ir tahereh.rohani@gmail.com; Research and Technology Center of Biomolecules, Faculty of Science, Ferdowsi University of Mashhad Mashhad 9177948974 Iran; Department of Chemical Engineering, Quchan University of Technology Quchan 94771-67335 Iran; Instituto de Ciencia de Materiales de Aragón (ICMA), Consejo Superior de Investigaciones Científicas (CSIC), Universidad de Zaragoza, CIBER-BBN C/Pedro Cerbuna 12 50009 Zaragoza Spain; School of Engineering, University of British Columbia Kelowna BC V1V 1V7 Canada; Department of Emergency Medicine, Faculty of Medicine, Ahvaz Jundishapur University Ahvaz Iran

## Abstract

Correction for ‘Sonochemical synthesis of polyoxometalate-stabilized gold nanoparticles for point-of-care determination of acetaminophen levels: preclinical study in an animal model’ by Tahereh Rohani Bastami *et al.*, *RSC Adv.*, 2020, **10**, 16805–16816, DOI: 10.1039/D0RA00931H.

The authors regret that there was an error in the sentence in lines 14–18 in the right column on page 16813 of the original article, in the sub-section “Sensing mechanism”. The text originally read: “To verify the mechanism of AuNPs aggregation in the presence of Ap, the following experiment was carried out: after the reaction nanosensor with AP, the final product was centrifuged and washed many times to purify and separate from the synthesis media.” This sentence should read: “To verify the mechanism of AuNPs aggregation in the presence of Ap, the following experiment was carried out: after the preparation of the nanosensor, the final product was centrifuged and washed many times to purify and separate from the synthesis media.”

The Royal Society of Chemistry apologises for these errors and any consequent inconvenience to authors and readers.

## Supplementary Material

